# Dominant Species Drive Biomass and Diversity Responses to Nutrient Inputs

**DOI:** 10.1002/ece3.73022

**Published:** 2026-02-17

**Authors:** Philip A. Fay, Anita C. Risch, Michael J. Aspinwall, Robert W. Heckman, Albina R. Khasanova, Lara G. Reichmann

**Affiliations:** ^1^ USDA‐ARS Grassland, Soil, and Water Research Lab Temple Texas USA; ^2^ Community Ecology Swiss Federal Institute for Forest, Snow and Landscape Research WSL Birmensdorf Switzerland; ^3^ Department of Integrative Biology University of Texas at Austin Austin Texas USA

**Keywords:** biodiversity, eutrophication, mesic grassland, multiple nutrient limitation, Nutrient Network, precipitation variability, tallgrass prairie

## Abstract

Global change is enriching terrestrial ecosystems with multiple nutrients and amplifying interannual variation in precipitation. Grassland productivity may be co‐limited by combinations of nitrogen (N), phosphorus (P), and potassium (K). How these nutrients may interact with each other or with varying precipitation to influence the contributions of dominant species and functional groups to aboveground net primary productivity (ANPP) and species diversity is rarely considered. We fertilized a mesic grassland for 5 years with all combinations of N, P, and K+ micronutrients in the first year (Kμ) to test which nutrients (1) limited ANPP and functional group biomass, (2) reordered dominant species and impacted plant species diversity, and (3) interacted with annual precipitation to influence these responses. Adding N and P together disproportionately increased ANPP, but adding both N and P or N and Kμ disproportionately increased forb biomass to account for nearly all (90%) of ANPP. Grass biomass was correlated with light availability, not nutrients, and legume biomass decreased with added N, with or without other nutrients. Nutrient combinations (mainly NP and NPKμ) causing the greatest increases in forb biomass and ANPP also resulted in replacement of dominant species by an annual forb and decreased species diversity (Shannon index), evenness, and species richness. Nutrient combinations (P, Kμ, PKμ) not increasing biomass favored dominance by C4 grasses and increased species richness. N effects on ANPP, species diversity, and richness were greater in years with higher annual precipitation. Annual precipitation interacted with all three nutrients to exert sometimes positive and sometimes negative feedback on the abundance of the most dominant species. Dominant species drive nutrient effects on community productivity and species diversity. An expanded definition of nutrient limitation incorporating constituent responses will improve understanding of anthropogenic nutrient inputs on ecosystem productivity and related ecosystem services.

## Graphical abstract

In a mesic grassland co‐limited by nitrogen and phosphorus, responses in plant community diversity and particularly of dominant species do not always correspond to responses in aboveground net primary productivity and functional group biomass production. Interactions involving a third nutrient, potassium, were particularly evident in reordering among dominant species.

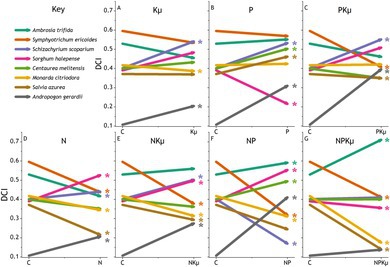

## 1 Introduction

Global change is enriching terrestrial ecosystems with nutrients, including nitrogen (N), phosphorus (P), and potassium (K), which co‐limit primary productivity (Elser et al. 2007; Mahowald et al. 2008; Sardans and Peñuelas 2015; Greaver et al. 2016). In grasslands, multiple nutrient fertilization experiments (Borer et al. 2014) show that aboveground net primary productivity (ANPP) is frequently co‐limited by multiple nutrients, often N and P (Fay et al. 2015). However, limitation by K may be more widespread than is commonly understood (Sardans and Peñuelas 2015; Liang et al. 2025), and gains in ANPP when limiting nutrients are supplied are typically larger in more mesic grasslands (Fay et al. 2025). Responses in the dominant species in plant communities are a key mechanism by which ANPP responds to changes in resource availability (Smith et al. 2009; Avolio et al. 2019; Wilfahrt et al. 2023). The responses of dominant species to inputs of co‐limiting nutrients remain poorly understood. Predicting dominant species responses is challenging because individual dominants may be limited by different combinations of nutrients than other constituents of the community such as subordinate species or forb, grass, and legume functional groups (Smith et al. 2009; Harpole et al. 2016; Pierre et al. 2016). Clarifying how the dominant species’ responses to co‐limiting nutrients align with those of ANPP is necessary to accurately predict how global change drivers impact ecosystem function (Smith et al. 2009; Komatsu et al. 2019).

Gains in ANPP with addition of co‐limiting nutrients are commonly accompanied by decreased community‐scale species diversity (Suding et al. 2005; Harpole et al. 2016; Koerner et al. 2016; Brown et al. 2022). If diversity losses concentrate in dominant species, community evenness would likely decline in concert with gains in ANPP, reinforced by decreased light availability (DeMalach et al. 2017; Harpole et al. 2017). However, if diversity losses concentrate in subordinate species or throughout the community, species richness would decline in concert with ANPP. However, Harpole et al. (2016) found that non‐limiting nutrients decreased species richness, whereas others noted that nutrients may limit ANPP but cause little or no diversity loss (Soons et al. 2017; DeMalach 2018; Carroll et al. 2022). The influence of nutrient combinations not limiting to ANPP (Fay et al. 2015; Carroll et al. 2022) on community level diversity is rarely considered. Identifying how both limiting and non‐limiting nutrient combinations impact dominant species hierarchies, subdominant species, and functional groups is necessary to understand their combined effects on community scale diversity, richness, and evenness.

Grasslands are also typically under water deficit. As a result, primary productivity in many grasslands is positively related to annual precipitation amount (Sala et al. 1988; Briggs and Knapp 1995; Huxman et al. 2004; Wilcox et al. 2016), which is becoming increasingly variable (Bosilovich et al. 2005; Padrón et al. 2020). Theory predicts that ANPP is increasingly limited by nutrient availability in years with higher annual precipitation (Huxman et al. 2004; Knapp et al. 2017). Fertilizing with limiting nutrients commonly increases the responsiveness of biomass to varying precipitation on both spatial and temporal scales (Eskelinen and Harrison 2015; Bharath et al. 2020; Fay et al. 2025). As a result, in wetter years biomass production may be more responsive to inputs of multiple co‐limiting nutrients. In turn, this would be expected to intensify the responses in plant communities to inputs of limiting and non‐limiting nutrients, including stronger functional group responses, amplified species losses, reduced evenness, and reordering of dominant species. Previous studies provide varying support for these predictions. For example, in some grasslands adding limiting nutrients yielded larger gains in biomass in wetter years (Bharath et al. 2020; Van Sundert et al. 2021; Carroll et al. 2022). In contrast, in a mesic grassland in the U.S. Central Plains where ANPP was strongly correlated with annual precipitation (Briggs and Knapp 1995), fertilizing with N and P eliminated that correlation by shifting the community from dominance by C4 grasses to forbs (Koerner et al. 2016). Increased annual precipitation may favor new dominant species regardless of change in overall richness, evenness, or functional group dominance (Collins et al. 2012; Eskelinen and Harrison 2015; Wilcox et al. 2016; Komatsu et al. 2019). How annual precipitation interacts with limiting or non‐limiting nutrients to affect plant community structure has received little attention.

Here, we fertilized a mesic grassland in the southern U.S. Central Plains with N, P, and K in all combinations, which is the definitive test to determine which nutrients limit ANPP (Gruner et al. 2008; Harpole et al. 2011; Fay et al. 2015). Grasslands in this region are commonly nutrient‐limited (Turner et al. 1997), experience substantial yearly variation in precipitation and frequent dry seasons (Changnon et al. 2002), and support a diverse flora dominated by warm‐season C4 grasses and numerous species of herbaceous dicots (forbs) (Freeman 1998). Specifically, we sought to identify which nutrients (1) limited ANPP and functional group biomass, (2) re‐ordered dominant species and impacted plant species diversity, and (3) interacted with annual precipitation to alter these responses. We hypothesized that (1) ANPP will be co‐limited by N and P, driven by responses in the most productive plant functional group; (2) Nutrient combinations limiting to ANPP will decrease community‐level species diversity, evenness, and species richness; (3) ANPP‐limiting and non‐limiting nutrient combinations will reorder dominant species; (4) responses to nutrient inputs will increase in wetter years. We tested these hypotheses with aboveground biomass production and plant community diversity data from 5 years of the experiment (2012–2016).

## 2 Materials and Methods

### 2.1 Study Site

This experimental site is part of the Nutrient Network globally distributed multiple nutrient addition experiment (temple.us; http://www.nutnet.org). Data from this site were included in previous multi‐site analyses (e.g., Hautier et al. 2014; Fay et al. 2015; Harpole et al. 2016). The study was established in a 3.3 ha never‐plowed remnant tallgrass prairie at Temple, TX, USA (31°05′ N, 97°20′ W), in the Blackland Prairie region of the U.S. Central Plains. Soils on this site are an upland silty clay Mollisol (Udorthentic Haplustolls) of the Austin series (Polley et al. 2012).

The climate at the study site is subtropical, falling in the transition between humid and sub‐humid zones. Long‐term mean annual precipitation (1981–2010) was 917 mm. Precipitation in this region falls primarily during May–June and September–October, with a pronounced July–August dry period. Temperatures range from a July–August mean maximum of 35°C to a December mean minimum of 2.9°C. The mean frost‐free period is ~250 days, from mid‐March to late November. Forbs accounted for most of the cover in the community, notably *Symphyotrichum ericoides* , *Ambrosia trifida* , *Monarda citriodora* , *Centaurea melitensis* , and *Salvia azurea* accompanied by the native C4 grasses *Schizachyrium scoparium* , *Andropogon gerardii* , and the non‐native C4 *Sorghum halepense* . In total, 45 species occurred during the period considered in this study, 2012–2016 (Table A1).

### 2.2 Site Management History

There is no known history of livestock grazing, haying, prescribed burning or other management removing aboveground plant biomass on the remnant since at least the early 1990s, including through the first years of the present study (2007–2011). As a result, woody species were increasingly abundant, and a management change was necessary to prevent loss of the native grassland community on this remnant to woody encroachment. Beginning in 2012, during late winter each February we removed all previous‐year senesced standing vegetation and litter from the entire remnant prairie using commercial haying equipment. We accomplished this on the study plots by cutting the vegetation at 5 cm height with a walk‐behind sickle‐bar mower and hand raking. During preliminary analyses we tested for nutrient effects on biomass and community diversity during 2008–2011, when standing dead biomass was not removed. Nutrient effects on biomass and diversity were generally weak and inconsistent with 2012–2016 patterns (Table A2, Tables 1 and 3), suggesting litter accumulation may have inhibited responses to applied nutrients as reported for other Nutrient Network sites (Seabloom et al. 2021). Thus, these years were not considered further.

### 2.3 Experimental Design

We applied the Nutrient Network standardized experimental design for factorial multiple nutrient treatments (Borer et al. 2014). In the pre‐treatment year, 2007, 24 5 × 5 m plots were arranged in a randomized complete block design with three replicates. This level of replication has proven adequate to identify site‐level nutrient treatment effects on aboveground biomass in grasslands across large geographic and climatic gradients (Fay et al. 2015, 2025). Walkways 1 m wide separated plots, and 3 m‐wide walkways separated blocks. Walkways were periodically mowed.

### 2.4 Fertilizer Treatments

In each block, plots were randomly assigned to receive one of 8 factorial combinations of N, P, and K with year 1 micronutrients (Kμ) fertilizers (N, P, Kμ, NP, NKμ, PKμ, NPKμ), including an unfertilized control (C). From 2008 to 2016, fertilizers were applied each April by manual broadcast.

Nutrient addition rates and sources were: (1) 10 g N m^−2^ year^−1^ as ammonium nitrate [NH_4_NO_3_]; (2) 10 g P m^−2^ year^−1^ as triple‐super phosphate [Ca(H_2_PO_4_)_2_]; and (3) 10 g K m^−2^ year^−1^ as potassium sulfate [K_2_SO_4_]. In 2008 only, K was combined with 100 g m^−2^ of a micronutrient mix (Kμ) containing Fe (15%), S (14%), Mg (1.5%), Mn (2.5%), Cu (1%), Zn (1%), B (0.2%) and Mo (0.05%; Scotts Micromax). Micronutrient applications were not repeated to avoid possible accumulation and toxicity. These are standard rates used at all Nutrient Network sites and chosen as likely to alleviate nutrient limitation in most grasslands (Borer et al. 2014).

### 2.5 Sampling Overview

We sampled the current year aboveground live plant biomass, species composition, and light levels at the base of the plant canopy using the Nutrient Network sampling protocol (Borer et al. 2014). Sampling was conducted in one dedicated 2.5 m × 2.5 m subplot in each plot. Sampling occurred twice per growing season, in early June and in early September at peak biomass, to represent the contributions of early and late season species to ANPP and plant species diversity.

### 2.6 Aboveground Biomass

We clipped at ground level all live current‐year plant growth rooted within two 0.1 m^−2^ strips (20 × 50 cm) placed in a designated 1 m^2^ portion of the sampling subplot. All sampled biomass represented current‐year production because previous year biomass was removed as described above. Current year live biomass estimates ANPP at this site because herbivory was minimal and little observable decomposition of current year growth occurred between sampling intervals (Knapp et al. 2007). The clip strips were moved at each sampling to avoid suppressing future production by repeatedly clipping the same location. Clipped biomass was sorted to forb, grass, and legume functional groups, dried at 60°C to constant mass and weighed to the nearest 0.01 g.

### 2.7 Plant Species Composition

The aerial cover was visually estimated to the nearest 1% for each plant species rooted in a dedicated 1 m^2^ quadrat within the sampling subplot. Total cover sums to > 100% because of vertical and horizontal overlap among individuals in the canopy. No destructive sampling took place in this quadrat to prevent disturbance to the canopy.

### 2.8 Canopy Light Levels

Sunlight intensity at the base of the canopy was measured in the species composition quadrat. Sunlight was measured with a ceptometer (SunScan; Delta‐T Devices Ltd., Cambridge, UK) at 10 cm above the soil surface across the diagonals of the quadrat.

### 2.9 Precipitation

Daily precipitation depth (mm) was measured with a tipping‐bucket gauge (TE525, Texas Electronics Inc., Dallas, TX, USA) and recorded by a data logger (CS1000, Campbell Scientific, Logan, UT, USA) at a site 0.5 km from the experiment except in 2012, when precipitation was measured at a station 1 km from the experimental site. Harvest year annual precipitation in mm year^−1^ was computed as the sum of daily precipitation between September biomass harvests. These amounts for 2012 through 2016, respectively, were 861, 687, 709, 979, and 1348 mm.

### 2.10 Data Reduction

Biomass, cover, and light measurements were reduced to a single value per plot each year. Aboveground biomass of grasses, forbs, and legumes was summed across the two clip samples on each sample date and scaled to g per m^−2^. The maximum of June or September functional group biomass was retained and summed to obtain ANPP. Similarly, the maximum of the June or September cover value for each species was retained for the calculation of species abundance and diversity metrics. The two diagonal ceptometer measurements were averaged, and the minimum of June or September light levels was retained.

### 2.11 Plant Species Abundance

For each plant species in each plot, we computed a frequency weighted species abundance, the Dominance Candidate Index (DCI, Equation 1) (Avolio et al. 2019):

(1) $$\mathrm{DCI}=\bar{X}\;\left(p_{i}+n_{i}/3\right)$$

We used plant species cover as a proxy for species abundance. DCI is the mean of two components: (1) the relative cover of plant species *i* (*p*_*i*_), the ratio of its absolute cover to total cover, and (2) the relative frequency of species *i* (*n*_*i*_), the number of plots out of three in each treatment in which species *i* appears. *DCI* is 0 if a species is absent from all three plots in a year. We identified a group of eight dominant species that were spatially and temporally common, defined as present in all years and in at least 60 of the 120 total plot‐years. The remaining 36 species were labeled subordinate species. The identified dominant species had the highest mean *DCI* values in the community, averaged across treatments and years (Table A1).

### 2.12 Community Diversity Indices

**FIGURE A1:**
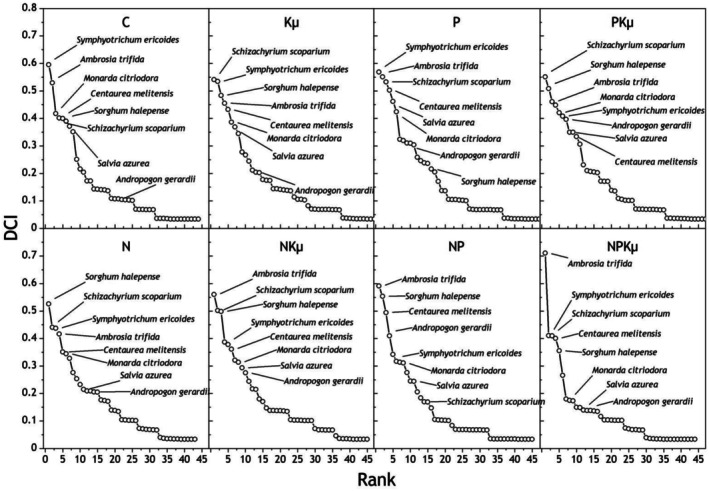
Rank—abundance curves from plant communities fertilized with single and multiple nutrient combinations of nitrogen (N), phosphorus (P), and potassium with year 1 micronutrients (Kμ) and unfertilized controls (C). The metric of abundance is the Dominance Candidate Index (DCI). The eight dominant species are labeled.

We computed three community‐level indices (Magurran 2013): plot‐level Shannon diversity; evenness, the inverse of dominance, which describes the equality of species abundances with a maximum value of 1 indicating all species are equally abundant; and species richness, the total number of species observed in species composition quadrats. Species richness was subdivided into the richness of dominant and subordinate species. Evenness was also characterized with species rank‐abundance curves constructed using the mean DCI for each species within treatments across years (Figure A1). Curves were fit with exponential decay functions.

### 2.13 Statistical Procedures

We tested the hypotheses by applying linear mixed models (SAS/STAT 12.3, SAS Institute 2013) to identify the independent and interactive effects of N, P, Kμ, and annual precipitation on 2012–2016 ANPP, functional group (grass, forb, and legume) biomass, ground‐level canopy light levels, Shannon index, evenness, total, dominant, and subordinate species richness, and DCI for each dominant species.

Response variables were scaled and centered ($\bar{X}$ = 0, SD = 1) in Proc STDIZE for model fitting. Species richness variables and dominant species DCIs were rank‐transformed because they did not meet distributional assumptions of linear mixed models.

We fit an analysis of covariance model in Proc MIXED to each response variable (Equation 2).

(2) $${\text{Response}}_{\mathrm{ij}}=\mu +{\text{nutrients}}_{i}+{\text{precip}}_{j}+\text{nutrients}\times {\text{precip}}_{\mathrm{ij}}+e_{\mathrm{ijk}}.$$

where *nutrients* represents all N, P, and Kμ main effects and 2‐way and 3‐way nutrient interactions, and *precip* represents annual precipitation amount on a harvest‐year basis. *F*‐statistics were computed using Type III sums of squares. Models included Block*N*P*Kμ as a plot‐level random effect. To account for potential lag effects between years from varying precipitation and the change in management, the random effects were modeled with a heterogeneous autoregressive order 1 (arh1) covariance structure, which accommodates differing variability between adjacent measurements. The arh1 structure improved model fit compared to structures assuming constant variability, indicated by a decrease of ~20 in Akaike's Information Criterion. In models fit to dominant species DCIs, we tested for significant treatment least squares means relative to control least squared means (lsmeans/pdiff statement).

Multiple nutrient interactions were identified as synergistic when the response to both nutrients was greater than the sum of the response to each nutrient individually, accompanied by a significant interaction term in the mixed model (Gruner et al. 2008; Harpole et al. 2011; Fay et al. 2025).

Nutrient combinations crossed with annual precipitation tested whether nutrient effects on response variables changed from wetter to drier years. For variables where annual precipitation or annual precipitation × nutrient interactions were significant, slopes were generated using the/solutions option. Response means and standard errors were graphed in original units (OriginPro 2025 v10.2.0.188, OriginLab Corporation, Northampton, MA, USA).

## 3 Results

### 3.1 Nutrients Limiting ANPP

**FIGURE 1:**
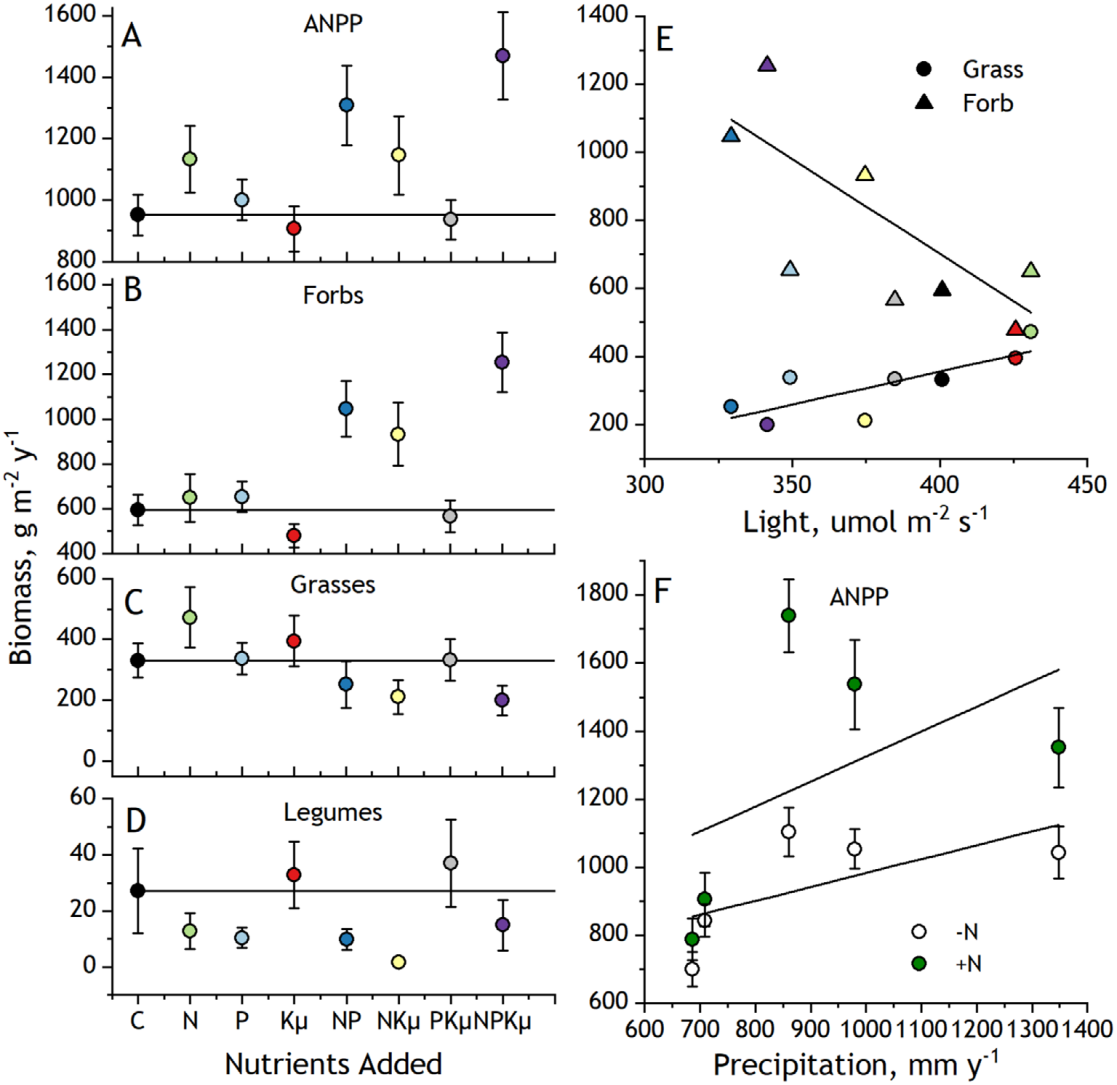
Means (±SE) responses over 5 years (2012–2016) of fertilizing plant communities with single and multiple nutrient combinations of nitrogen (N), phosphorus (P), potassium with year 1 micronutrients (Kμ), and unfertilized controls (C). (A) Aboveground net primary production (ANPP), (B) forb biomass, (C) grass biomass, (D) legume biomass, (E) associations of mean forb and mean grass biomass with mean ground‐level canopy light intensity with linear regression fits, (F) ANPP in relation to harvest year annual precipitation averaged across treatments containing N (+N: N, NP, NKμ, NPKμ) and lacking N (−N: C, P, Kμ, PKμ) with linear regression fits. See Table 1 for accompanying linear mixed‐model analysis.

As hypothesized, adding N and P disproportionately increased ANPP (Figure 1A). ANPP increased 15% when fertilized with N alone, 6% with P alone, but 33% with NP (N*P: *p* = 0.05, Table 1), an indication of synergistic co‐limitation. There was no indication that fertilization with Kμ interacted with N and P to affect ANPP (0.14 < *p* < 0.58, Table 1). The gains in ANPP with added N and P aligned closely with responses of forb biomass, which accounted for the majority (62%) of control plot ANPP. Adding NP increased forb biomass 76% averaged across levels of Kμ compared to unfertilized plots; forb biomass increased only ~10% when either N or P were added individually (N*P *p* = 0.0085, Table 1). Unexpectedly, adding NKμ increased forb biomass to a similar degree as with NP fertilization (N*Kμ *p* = 0.0017, Figure 1B, Table 1), overcoming a decrease in forb biomass when Kμ alone was added. Notably, adding NPKμ yielded over twice the forb biomass of controls, accounting for 90% of ANPP. Mean forb biomass in the nutrient treatments correlated with decreased canopy light intensity (*R* ^2^ = 0.51, *p* < 0.03; Figure 1E).

**TABLE 1:** Analysis of covariance *F*‐statistics, degrees of freedom (dfs), and *p*‐values from linear mixed models fit to aboveground net primary productivity (ANPP) and forb, grass, and legume biomass (Figure 1).

Mixed‐model effects	ANPP		Forb biomass		Grass biomass		Legume biomass	
	*F*(dfs)	*p*	*F*(dfs)	*p*	*F*(dfs)	*p*	*F*(dfs)	*p*
N	28.2 (1, 16)	**< 0.0001**	48.2 (1, 16)	**< 0.0001**	3.4 (1, 16)	0.0841	7.1 (1, 16)	**0.0170**
P	9.0 (1, 16)	**0.0084**	23.3 (1, 16)	**0.0002**	0.5 (1, 16)	0.5073	1.0 (1, 16)	0.3427
N*P	4.5 (1, 16)	**0.0504**	9.0 (1, 16)	**0.0085**	0.0 (1, 16)	0.8982	2.3 (1, 16)	0.1470
Kμ	0.3 (1, 16)	0.5762	4.7 (1, 16)	**0.0454**	2.2 (1, 16)	0.1581	0.2 (1, 16)	0.6593
N*Kμ	2.4 (1, 16)	0.1435	15.5 (1, 16)	**0.0012**	2.3 (1, 16)	0.1522	1.8 (1, 16)	0.1963
P*Kμ	1.5 (1, 16)	0.2404	0.0 (1, 16)	0.9910	0.0 (1, 16)	0.8345	0.4 (1, 16)	0.5204
N*P*Kμ	2.0 (1, 16)	0.1813	0.1 (1, 16)	0.7081	1.1 (1, 16)	0.3121	0.7 (1, 16)	0.4186
AP	57.7 (1, 88)	**< 0.0001**	8.1 (1, 88)	**0.0055**	8.3 (1, 88)	**0.0050**	6.1 (1, 88)	**0.0155**
AP*N	6.5 (1, 88)	**0.0124**	2.8 (1, 88)	0.0981	1.3 (1, 88)	0.2656	3.4 (1, 88)	0.0668
AP*P	0.2 (1, 88)	0.6345	0.1 (1, 88)	0.7551	0.0 (1, 88)	0.9845	0.2 (1, 88)	0.6629
AP*N*P	0.5 (1, 88)	0.4925	0.1 (1, 88)	0.7870	2.9 (1, 88)	0.0905	0.1 (1, 88)	0.7484
AP*Kμ	0.3 (1, 88)	0.5721	0.3 (1, 88)	0.5867	0.0 (1, 88)	0.9973	0.0 (1, 88)	0.8702
AP*N*Kμ	0.1 (1, 88)	0.7296	1.3 (1, 88)	0.2537	1.6 (1, 88)	0.2061	0.6 (1, 88)	0.4288
AP*P*Kμ	0.0 (1, 88)	0.9486	0.0 (1, 88)	0.9707	0.8 (1, 88)	0.3800	0.1 (1, 88)	0.7432
AP*N*P*Kμ	0.2 (1, 88)	0.6673	0.0 (1, 88)	0.9861	0.1 (1, 88)	0.7602	1.0 (1, 88)	0.3237

*Note:* Models tested effects of fertilizing communities with single and multiple nutrient combinations of nitrogen (N), phosphorus (P), and potassium with year 1 micronutrients (Kμ) and their interactions with annual precipitation on a harvest year basis (AP) during 2012–2016. Bolded *p*‐values denote *p* < 0.055.

Grass biomass responses to fertilization were not aligned with those of ANPP, with no significant response to any combination of N, P, or Kμ inputs (0.08 < *p* < 0.90, Table 1). Trends in grass biomass responses to fertilization offset responses in forbs, particularly in treatments combining N with P or Kμ (Figure 1C). Instead, mean grass biomass in the fertilization treatments increased with mean light intensity in the canopy (*R* ^2^ = 0.56, *p* < 0.02, Figure 1E), suggesting that grass biomass was more coupled to forb‐driven changes in light availability than to fertilizer treatments.

Legumes accounted for only 3%–4% of ANPP and averaged across other treatments, legume biomass decreased with N fertilization (N main effect *p* = 0.017, Table 1, Figure 1D), but no other main or interactive effects of fertilization treatments were detected (0.15 < *p* < 0.66).

### 3.2 Reordering of Dominant Species

**FIGURE 2:**
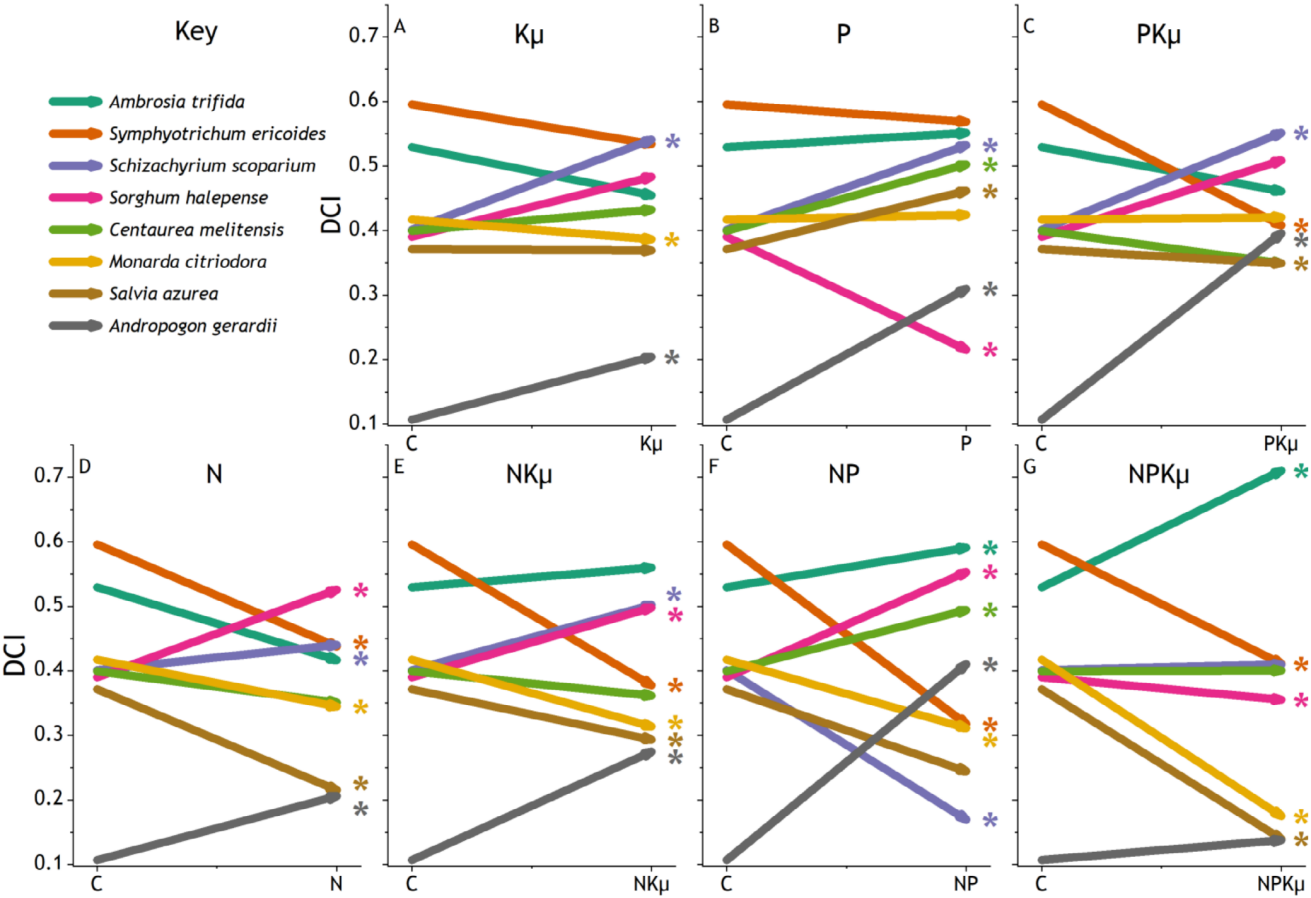
Vector plots of responses of the eight dominant species to fertilization of plant communities with single and multiple nutrient combinations of nitrogen (N), phosphorus (P), potassium with year 1 micronutrients (Kμ), and unfertilized controls (C). Each vector represents the Dominance Candidate Index (DCI) for a species. The tail of each vector is located at the mean DCI in control plots and the head at the mean DCI of the treatment. Asterisks near the vector head indicate significant (*p* < 0.05) change in DCI from the control. See Figure A1 for the complete rank abundance curves including the subordinate species.

**FIGURE 3:**
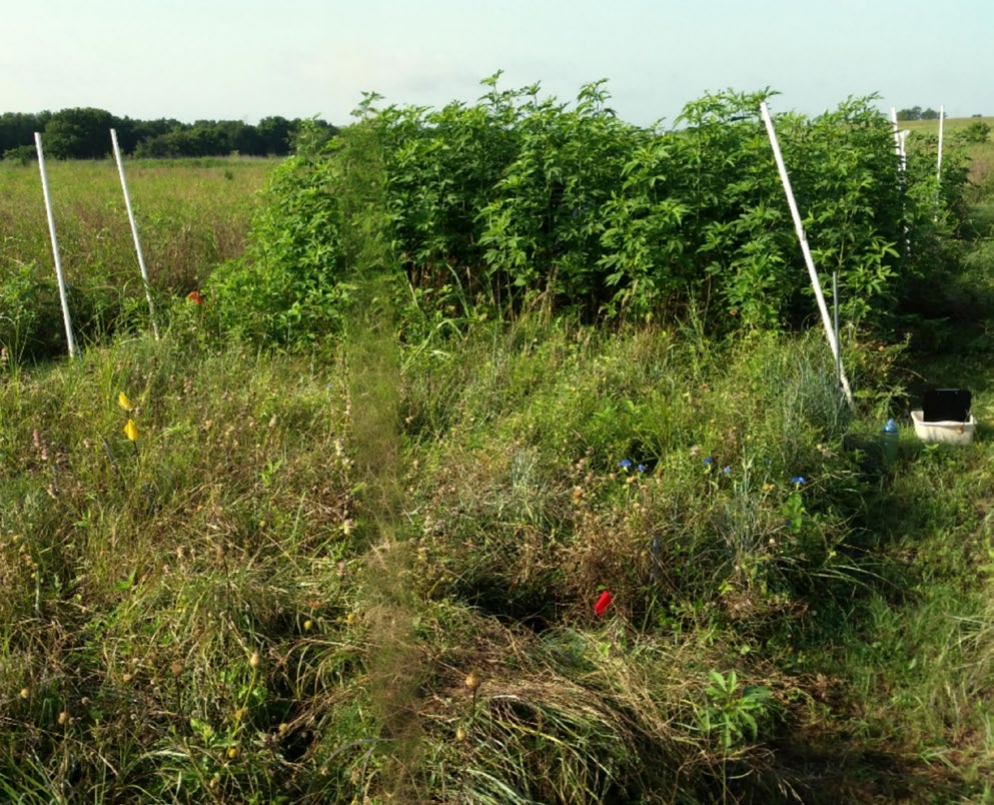
Example of structural dominance of the plant community by an annual forb ( *Ambrosia trifida* ) in plots receiving all three nutrients, N, P, and Kμ. The foreground plot was an unfertilized control. The image was edited to remove a foreground obstacle.

**FIGURE A2:**
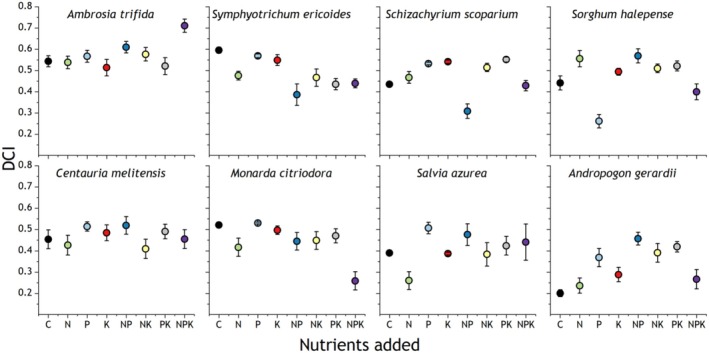
Means (±SE) for the Dominance Candidate Index (DCI) for the eight dominant species over 5 years (2012–2016) of fertilizing communities with single and multiple nutrient combinations of nitrogen (N), phosphorus (P), potassium with year 1 micronutrients (Kμ), and unfertilized controls (C).

Abundances of the eight dominant species were strongly influenced by many combinations of added N, P, and Kμ (0.0001 < *p* < 0.04, Table 2, Figure A2), both those that limited ANPP and those that did not. Nutrient combinations that did not increase ANPP or forb biomass (Figure 1A,B), Kμ, P and PKμ, caused convergence of DCI among the dominant species. DCI increased for two C4 grasses, *Andropogon gerardii* and *Schizachyrium scoparium* , which became co‐dominant with or replaced the forb *Symphyotrichum ericoides* (Figure 2A–C). In contrast, nutrient combinations that increased forb biomass and ANPP, N alone or with P and Kμ, caused divergence of DCI among the dominant species primarily because of decreases in most forbs (Figure 2D–F). With N alone, forb losses left the C4 grass *Sorghum halepense* dominant (Figure 2D). Adding N with Kμ or P resulted in co‐dominance by the annual forb *Ambrosia trifida* , and adding NPKμ strongly suppressed other dominant species (Figure 2G) leaving *A. trifida* to structurally dominate the plant canopy (Figure 3).

**TABLE 2:** Analysis of covariance *F*‐statistics, degrees of freedom (dfs), and *p*‐values linear mixed models fit to the Dominance Candidate Index for the eight dominant species (Figure 2).

Mixed‐model effects	*Ambrosia trifida*		*Symphyotrichum ericoides*		*Schizachyrium scoparium*		*Sorghum halepense*	
	*F* (dfs)	*p*	*F* (dfs)	*p*	*F* (dfs)	*p*	*F* (dfs)	*p*
N	47.9 (1, 16)	**< 0.0001**	41.7 (1, 16)	**< 0.0001**	22.3 (1, 16)	**0.0002**	29.6 (1, 16)	**< 0.0001**
P	28.5 (1, 16)	**< 0.0001**	4.8 (1, 16)	**0.0429**	17.0 (1, 16)	**0.0008**	26.6 (1, 16)	**< 0.0001**
N*P	19.9 (1, 16)	**0.0004**	1.9 (1, 16)	0.1917	78.9 (1, 16)	**< 0.0001**	0.6 (1, 16)	0.4629
Kμ	5.1 (1, 16)	**0.0385**	9.6 (1, 16)	**0.0068**	27.7 (1, 16)	**< 0.0001**	1.3 (1, 16)	0.2711
N*Kμ	9.5 (1, 16)	**0.0072**	7.3 (1, 16)	**0.0158**	1.3 (1, 16)	0.2733	48.0 (1, 16)	**< 0.0001**
P*Kμ	1.5 (1, 16)	0.2314	0.0 (1, 16)	0.9811	3.7 (1, 16)	0.0717	0.4 (1, 16)	0.5251
N*P*Kμ	0.4 (1, 16)	0.5305	5.4 (1, 16)	**0.0335**	5.0 (1, 16)	**0.0407**	18.9 (1, 16)	**0.0005**
AP	249.2 (1, 88)	**< 0.0001**	23.3 (1, 88)	**< 0.0001**	10.3 (1, 88)	**0.0019**	21.3 (1, 88)	**< 0.0001**
AP*N	0.4 (1, 88)	0.5157	1.5 (1, 88)	0.2307	12.3 (1, 88)	**0.0007**	3.0 (1, 88)	0.0844
AP*P	0.2 (1, 88)	0.6405	0.0 (1, 88)	0.8790	12.2 (1, 88)	**0.0007**	1.5 (1, 88)	0.2179
AP*N*P	3.1 (1, 88)	0.0805	18.1 (1, 88)	**< 0.0001**	4.4 (1, 88)	**0.0398**	4.6 (1, 88)	**0.0354**
AP*Kμ	0.2 (1, 88)	0.6741	24.5 (1, 88)	**< 0.0001**	0.0 (1, 88)	0.9831	10.5 (1, 88)	**0.0017**
AP*N*Kμ	5.8 (1, 88)	**0.0183**	3.4 (1, 88)	0.0669	5.2 (1, 88)	**0.0247**	27.8 (1, 88)	**< 0.0001**
AP*P*Kμ	1.1 (1, 88)	0.2977	10.5 (1, 88)	**0.0017**	2.9 (1, 88)	0.0936	0.8 (1, 88)	0.3729
AP*N*P*Kμ	0.0 (1, 88)	0.8377	0.5 (1, 88)	0.4720	0.1 (1, 88)	0.8231	2.5 (1, 88)	0.1187

	*Centaurea melitensis*		*Monarda citriodora*		*Salvia azurea*		*Andropogon gerardii*	
N	8.2 (1, 16)	**0.0113**	84.7 (1, 16)	**< 0.0001**	78.6 (1, 16)	**< 0.0001**	1.4 (1, 16)	0.2486
P	20.2 (1, 16)	**0.0004**	0.1 (1, 16)	0.7661	0.1 (1, 16)	0.7871	67.3 (1, 16)	**< 0.0001**
N*P	4.1 (1, 16)	0.0590	19.5 (1, 16)	**0.0004**	2.8 (1, 16)	0.1127	35.2 (1, 16)	**< 0.0001**
Kμ	8.5 (1, 16)	**0.0100**	13.6 (1, 16)	**0.0020**	35.6 (1, 16)	**< 0.0001**	2.4 (1, 16)	0.1388
N*Kμ	0.4 (1, 16)	0.5312	0.2 (1, 16)	0.6396	3.6 (1, 16)	0.0762	45.2 (1, 16)	**< 0.0001**
P*Kμ	20.2 (1, 16)	**0.0004**	0.1 (1, 16)	0.7206	77.5 (1, 16)	**< 0.0001**	36.9 (1, 16)	**< 0.0001**
N*P*Kμ	1.9 (1, 16)	0.1869	3.7 (1, 16)	0.0730	3.0 (1, 16)	0.1019	30.6 (1, 16)	**< 0.0001**
AP	86.8 (1, 88)	**< 0.0001**	98.9 (1, 88)	**< 0.0001**	25.8 (1, 88)	**< 0.0001**	17.8 (1, 88)	**< 0.0001**
AP*N	0.0 (1, 88)	0.8523	13.4 (1, 88)	**0.0004**	37.4 (1, 88)	**< 0.0001**	105.5 (1, 88)	**< 0.0001**
AP*P	9.1 (1, 88)	**0.0033**	6.3 (1, 88)	**0.0138**	1.6 (1, 88)	0.2105	1.2 (1, 88)	0.2850
AP*N*P	0.9 (1, 88)	0.3589	1.6 (1, 88)	0.2148	9.7 (1, 88)	**0.0024**	10.0 (1, 88)	**0.0022**
AP*Kμ	2.8 (1, 88)	0.1005	0.2 (1, 88)	0.6779	7.8 (1, 88)	**0.0065**	6.5 (1, 88)	**0.0126**
AP*N*Kμ	12.8 (1, 88)	**0.0006**	5.5 (1, 88)	**0.0217**	1.2 (1, 88)	0.2781	4.0 (1, 88)	**0.0491**
AP*P*Kμ	16.6 (1, 88)	**0.0001**	2.2 (1, 88)	0.1420	31.6 (1, 88)	**< 0.0001**	3.5 (1, 88)	0.0654
AP*N*P*Kμ	14.7 (1, 88)	**0.0002**	0.0 (1, 88)	0.8522	11.5 (1, 88)	**0.0011**	81.6 (1, 88)	**< 0.0001**

*Note:* Models tested effects of fertilizing with combinations of nitrogen (N), phosphorus (P), and potassium with year 1 micronutrients (Kμ) and their interactions with harvest‐year annual precipitation (AP) during 2012–2016. Bolded *p*‐values denote *p* < 0.055.

### 3.3 Responses in Community‐Level Diversity

**FIGURE 4:**
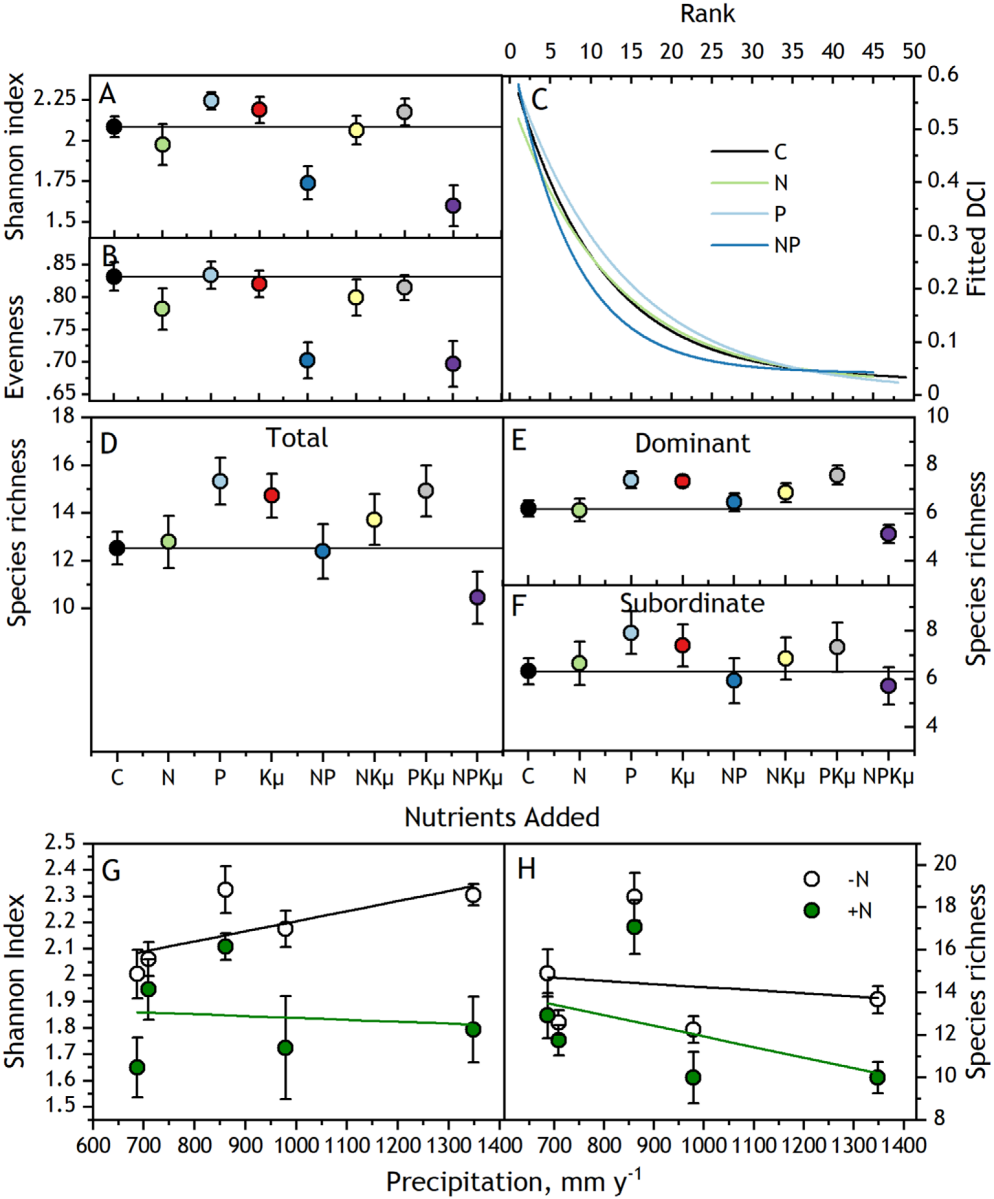
Means (±SE) responses over 5 years (2012–2016) of fertilizing plant communities with single and multiple nutrient combinations of nitrogen (N), phosphorus (P), potassium with year 1 micronutrients (Kμ), and unfertilized controls (C). (A) Species diversity (Shannon index), (B) Evenness, (C) Species rank vs. abundance (Dominance Candidate Index, DCI) curves fit across levels of Kμ for control, N, P, and NP treatments. Data underlying these curves are shown in Figure A3. (D) Species richness (total), (E) Richness of dominant species, (F) Richness of subordinate species. (G) Shannon index and (H) Species richness averaged across levels of P and Kμ in relation to harvest year annual precipitation, with linear regression fits. See Table 3 for accompanying linear mixed model analyses.

As hypothesized, N and P, the combination co‐limiting to ANPP, decreased community‐level species diversity. Fertilizing with NP caused a disproportionately decreased Shannon diversity index (Figure 4A; NP interaction, *p* = 0.01, Table 3). Adding NPKμ caused the greatest decrease (Figure 4A), coinciding with the highest ANPP, forb biomass, and *A. trifida* dominance. In contrast, the individual nutrients caused little change in Shannon diversity (N, 6% decrease; P, 3% increase; Figure 4A). Kμ did not interact with N or P to influence the Shannon index (0.25 < *p* < 0.93, Table 3). Evenness responses to inputs of N, P, and Kμ matched those of ANPP and the Shannon index, decreasing disproportionately with added NP (NP interaction, *p* = 0.048, Table 3) but not influenced by added Kμ (0.77 < *p* < 0.98, Table 3). Decreases in evenness with added N and P were corroborated by shifts in rank abundance curves. Fertilization with NP made rank abundance curves more concave than curves for controls, indicating greater dominance by more abundant species (Figure 4C). In contrast, fertilization with P made curves less concave, indicating more equal abundances among species. Fertilizing with N and not P resulted in a curve like the control curve, suggesting N had little effect on the hierarchy of species abundances.

**TABLE 3:** Analysis of covariance *F*‐statistics, degrees of freedom (dfs), and *p*‐values from linear mixed models fit to the Shannon index of species diversity, evenness, species richness, and its subgroups, dominant, and subordinate species richness (Figure 4).

Mixed‐model effects	Shannon index		Evenness		Species richness		Dominant richness		Subordinate richness	
	*F* (dfs)	*p*	*F* (dfs)	*p*	*F* (dfs)	*p*	*F* (dfs)	*p*	*F* (dfs)	*p*
N	17.9 (1, 16)	**0.0006**	15.2 (1, 16)	**0.0013**	7.1 (1, 16)	**0.0168**	12.4 (1, 16)	**0.0029**	1.3 (1, 16)	0.2751
P	1.6 (1, 16)	0.2191	2.6 (1, 16)	0.1244	0.0 (1, 16)	0.9357	0.4 (1, 16)	0.5250	0.0 (1, 16)	0.8716
N*P	8.5 (1, 16)	**0.0103**	4.6 (1, 16)	**0.0480**	7.7 (1, 16)	**0.0133**	5.2 (1, 16)	**0.0364**	1.8 (1, 16)	0.2002
Kμ	0.0 (1, 16)	0.8580	0.1 (1, 16)	0.7967	0.6 (1, 16)	0.4567	1.0 (1, 16)	0.3347	0.2 (1, 16)	0.6360
N*Kμ	0.0 (1, 16)	0.9272	0.0 (1, 16)	0.9646	0.8 (1, 16)	0.3798	1.4 (1, 16)	0.2470	0.0 (1, 16)	0.9601
P*Kμ	1.4 (1, 16)	0.2495	0.1 (1, 16)	0.7662	3.2 (1, 16)	0.0917	8.7 (1, 16)	**0.0094**	0.2 (1, 16)	0.6469
N*P*Kμ	0.0 (1, 16)	0.8468	0.0 (1, 16)	0.9802	0.0 (1, 16)	0.8609	0.8 (1, 16)	0.3966	0.3 (1, 16)	0.6048
AP	0.8 (1, 88)	0.3839	14.1 (1, 88)	**0.0003**	2.1 (1, 88)	0.1514	2.5 (1, 88)	0.1152	3.3 (1, 87)	0.0710
AP*N	3.7 (1, 88)	**0.0575**	0.9 (1, 88)	0.3440	4.7 (1, 88)	**0.0321**	3.6 (1, 88)	0.0606	2.1 (1, 87)	0.1473
AP*P	0.4 (1, 88)	0.5174	0.0 (1, 88)	0.8777	0.1 (1, 88)	0.7878	1.1 (1, 88)	0.3040	0.1 (1, 87)	0.8089
AP*N*P	0.0 (1, 88)	0.9040	0.1 (1, 88)	0.8184	0.0 (1, 88)	0.9653	0.9 (1, 88)	0.3478	0.2 (1, 87)	0.6879
AP*Kμ	0.8 (1, 88)	0.3593	0.4 (1, 88)	0.5405	3.5 (1, 88)	0.0659	1.7 (1, 88)	0.1955	0.7 (1, 87)	0.3930
AP*N*Kμ	0.0 (1, 88)	0.9689	0.4 (1, 88)	0.5293	0.1 (1, 88)	0.8051	0.1 (1, 88)	0.7029	0.2 (1, 87)	0.6280
AP*P*Kμ	0.3 (1, 88)	0.5684	0.8 (1, 88)	0.3727	0.2 (1, 88)	0.6602	0.0 (1, 88)	0.8279	0.2 (1, 87)	0.6597
AP*N*P*Kμ	0.4 (1, 88)	0.5242	0.1 (1, 88)	0.7609	0.1 (1, 88)	0.7780	0.0 (1, 88)	0.9850	0.2 (1, 87)	0.6726

*Note:* Models tested effects of fertilizing communities with single and multiple nutrient combinations of nitrogen (N), phosphorus (P), and potassium with year 1 micronutrients (Kμ) and their interactions with annual precipitation on a harvest year basis (AP) during 2012–2016. Bolded *p*‐values denote *p* < 0.055.

Species richness also expressed an interaction between N and P (NP interaction *p* = 0.013, Table 3). However, contrary to the hypothesis, this interaction emerged from a different combination of responses to added N, P, and Kμ, coinciding more with dominant species responses than with ANPP or forb biomass. Species richness decreased only in the NPKμ treatment (Figure 4D), coinciding with strong *A. trifida* dominance. In contrast, species richness increased with added P, Kμ, PKμ, and to a lesser extent NKμ, coinciding with C4 grass dominance. Species richness did not respond to added N or NP. This combination of fertilization effects on species richness was driven by the richness of dominant species (Figure 4E), which were influenced by NP (*p* = 0.02) and PK interactions (*p* = 0.0094, Table 3). Richness of subordinate species was not influenced by the fertilizer treatments (0.20 < *p* < 0.96, Table 3).

### 3.4 Nutrient Interactions With Annual Precipitation

**FIGURE 5:**
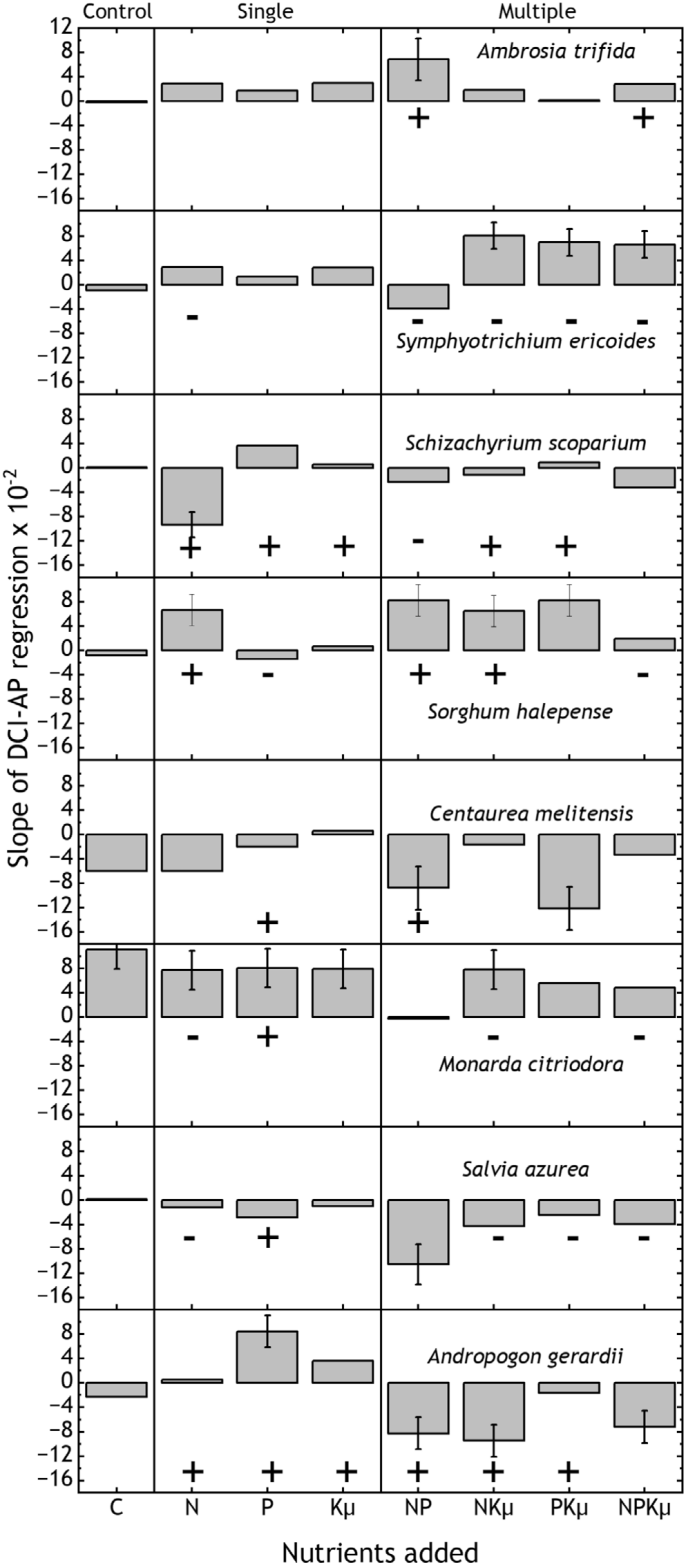
Slopes (±SE) of the DCI‐annual precipitation relationships in the eight dominant species in communities fertilized with single and multiple nutrient combinations of nitrogen (N), phosphorus (P), and potassium with year 1 micronutrients (Kμ), and unfertilized controls. Slopes with error bars were significantly different from zero. Symbols ‘+’ or ‘−’ denote the direction of significant treatment responses of mean DCI (Figure 2), to highlight whether the direction and significance of slopes correspond to those of mean treatment effects.

Variation in annual precipitation amount had pronounced effects on nutrient‐induced reordering among the individual dominant species. All had at least one, and usually several significant interactions of AP with various combinations of all three nutrients (Table 2). Species DCIs were correlated with annual precipitation more often in the multiple nutrient treatments (44% of 32 treatment‐species combinations) than in single nutrient treatments (25% of treatment‐species combinations, Figure 5). In most multiple nutrient treatments, the annual precipitation‐DCI correlations had positive slopes for *Symphyotrichum ericoides* , *Sorghum halepense* , and *Monarda citriodora*, indicating larger responses in wetter years. In contrast, for *Schizachyrium scoparium* , *Centaurea melitensis* , *Salvia azurea* , and *Andropogon gerardii* , the annual precipitation‐DCI correlations in multiple nutrient treatments were predominantly negative or neutral. For some species, slopes of single nutrient treatments sometimes aligned with multiple nutrient slopes ( *Sorghum halepense* , *Monarda citriodora* ) and did not in others ( *Schizachyrium scoparium* , *Andropogon gerardii*). Also, the sign of the annual precipitation‐DCI slopes sometimes opposed the mean treatment effect (e.g., *Symphyotrichum ericoides* , *Monarda citriodora* , and *Andropogon gerardii* ), indicating the variation in annual precipitation weakened the treatment effect. In other cases, the sign of the slope reinforced the treatment effect (N and NP effects for *Sorghum halepense* and single P effects for *Andropogon gerardii* ).

All biomass fractions and evenness were significantly related to precipitation overall (0.0001 < *p* < 0.016). However, only a few significant precipitation‐ nutrient interactions were identified. In these cases, responses were greater in wet years as hypothesized. With increasing annual precipitation, added N caused greater increases in ANPP and greater decreases in Shannon index and species richness (Figure 4G,H; 0.02 < *R* ^2^ < 0.51; AP × N 0.057 > *p* > 0.012, Tables 1 and 3).

## 4 Discussion

Ongoing enrichment of ecosystems with potentially limiting plant nutrients, combined with variability in annual precipitation are predicted to have strong effects on grassland ANPP (Elser et al. 2007; Mahowald et al. 2008; Sardans and Peñuelas 2015; Greaver et al. 2016) and plant species diversity (Suding et al. 2005; Harpole et al. 2016; Koerner et al. 2016; Brown et al. 2022). Dominant plant species play a central role in these responses (Smith et al. 2009; Avolio et al. 2019; Wilfahrt et al. 2023). Identifying how dominant species responses align with responses in other constituents of the community is key to understanding community‐level responses in species diversity and ANPP. Fertilizing with factorial combinations of N, P, and Kμ demonstrated that adding N and P together synergistically increased ANPP in this grassland because combinations of N, P, and Kμ extensively reordered dominant species, yielding increases in forb biomass in nutrient combinations where a single fast growing species replaced other dominants and decreased species diversity. These findings highlight a critical role of nominally non‐limiting nutrients, whose role may not be apparent in ANPP responses.

### 4.1 Nutrient Limitation of ANPP

N and P are broadly important nutrients across terrestrial ecosystems (Elser et al. 2007). The finding that N and P synergistically co‐limited ANPP in this mesic grassland aligns with long‐held understanding of plant biology and ecosystem function. N limitation has long been recognized (Hooper and Johnson 1999). N and P individually limited or jointly co‐limited ANPP in 60%–70% of global grasslands, including other mesic grasslands, and synergistic co‐limitation of ANPP by N and P occurred in 15%–20% of grasslands (Fay et al. 2015, 2025). Co‐limitation of grassland ANPP by N and P is common for several reasons. Plant demand for both elements is high. N is a constituent of the key enzyme catalyzing photosynthetic carbon dioxide uptake, and P is abundant in cell membranes and energy transfer molecules (Mahowald et al. 2008; Greaver et al. 2016). Supply of N and P varies depending on soils, climate, and management (Walker and Syers 1976; Vitousek et al. 2010), and for N, the size and turnover rates of soil organic matter pools (Franzluebbers et al. 2019). P is primarily found in inorganic forms, and its availability to some species depends on mycorrhizal symbionts (Johnson et al. 2010).

Although we did not resolve significant effects of adding Kμ on ANPP in this grassland, Kμ nonetheless played a key role, combining with N to increase forb biomass production (Figure 1B). This effect was not resolved in ANPP because forb responses were offset by declines in grass biomass likely caused by increased competition for light (Harpole et al. 2017; Wilfahrt et al. 2020). These findings indicate that Kμ limited forb biomass production. N and P also combined to increase the contribution of forb biomass to ANPP, as reported in other mesic grasslands (Avolio et al. 2014), although other studies found that grass responses predominate (Van Sundert et al. 2021; Mohanbabu et al. 2024). The predominant role of forbs follows from their relatively high initial dominance (60% of total aboveground biomass) in unfertilized plots (Suding et al. 2005; Chen et al. 2020). In this grassland, the dominant forbs possessed traits favored by fertilization, including early, rapid growth and tall stature, disadvantaging many shorter or later growing C_4_ grasses and legumes (Eskelinen and Harrison 2015). We did not resolve significant effects of N, P, or Kμ fertilization on legumes. Legumes are often favored where N is limiting (Tognetti et al. 2021), where the energetic costs of biological N fixation return greater relative benefits in plant growth. However, in this study, legumes constituted a small fraction of total biomass and likely experienced intensified competition for light from the non‐legume forbs.

### 4.2 Reordering of Dominant Species

N, P, and Kμ addition caused distinct changes in dominant species hierarchies coinciding with responses in forb biomass. The outcome of reordering hinged on whether N was included in the treatment. For example, adding P, Kμ, or PKμ, which had little effect on forb biomass or ANPP, increased the abundance of two productive C4 grasses, *Andropogon gerardii* and *Schizachyrium scoparium* , which are dominant species throughout tallgrass prairie (Hartnett and Fay 1998). In contrast, the largest forb biomass gains occurred with added NP, NKμ, or NPKμ, where most dominant forbs decreased, replaced by the exotic grass *Sorghum halepense* and especially the annual forb *Ambrosia trifida* . This reordering of dominant species amplified forb biomass production and contributed to the response in overall productivity (ANPP) (Brown et al. 2022). Underlying mechanisms likely included changing competitive interactions (Mohanbabu et al. 2024), and species‐specific nutrient requirements (Spohn 2024) or acquisition strategies (Cheaib et al. 2025).

### 4.3 Responses in Community‐Level Diversity

Decreases in Shannon diversity and evenness corresponded to the increases in ANPP and forb biomass from fertilizing with N and P. This result contrasts with reports of increased evenness following fertilization with N and P (Avolio et al. 2014), or no response averaged across multiple grasslands (Seabloom et al. 2021). The correspondence between evenness and ANPP in this grassland suggests a mechanistic link between evenness and biomass production as predicted by the mass ratio hypothesis (Grime 1998). Rank abundance curves corroborated this link, showing that adding NP increased dominant species abundances at the expense of subordinate species, impacting community structure as predicted by theory (Smith et al. 2009; Avolio et al. 2019). Evenness decreased when species in niches defined by the intersection of high light and lower N and P were lost, and the remaining species suited to low light and higher nutrients increased in abundance. These findings align with the well‐known role of N availability for grassland species diversity (Suding et al. 2005; Clark and Tilman 2008; Isbell et al. 2013; Koerner et al. 2016; Soons et al. 2017) and that adding multiple nutrients increases diversity loss (Harpole et al. 2016).

In contrast, species richness responses to nutrient treatments were distinct from biomass responses. Richness increased with added non‐limiting combinations P, Kμ, and PKμ, was unchanged with some added limiting combinations (N, NP), and decreased only when all three nutrients (NPKμ) were added. These findings contrast with other reports that enrichment of grasslands with N and P or with P and K decreased species richness (Avolio et al. 2014; van Dobben et al. 2017; Ward et al. 2017). The responses of species richness to limiting and non‐limiting nutrient combinations aligned with coordinated responses in the richness and abundance of the dominant species rather than of subordinate species or ANPP and forb biomass, and highlight the important role of individual dominant species responses in determining overall species richness. Increased abundance of some species (e.g., C4 grasses *Andropogon gerardii* and *Schizachyrium scoparium* ) when Kμ was added was unexpected. K is often abundant, especially in clay soils (Øgaard et al. 2001) such as those at this study site. In addition, the concentration of K is tightly regulated in plant tissues compared to N or P (Sardans and Peñuelas 2015) because of its critical role in metabolite transport and water balance.

### 4.4 Nutrient Interactions With Precipitation

As predicted, adding N but not P or Kμ resulted in larger gains in ANPP and decreases in Shannon index and species richness in wetter years, which suggests plant demand for nutrients was increased (Flores‐Moreno et al. 2016; Knapp et al. 2017; Bharath et al. 2020; Van Sundert et al. 2021; Carroll et al. 2022). Interactions between one or more nutrient combinations and annual precipitation influenced the abundances of all eight dominant species. For some dominant species, variation in annual precipitation exerted a negative feedback on responses to added nutrients because annual precipitation–DCI slopes were the opposite of the mean nutrient effects (e.g., *A. gerardii* , *S. ericoides*, *M. citriodora* ). For other species, there was positive feedback. Notably, in *Sorghum halepense* and especially *Ambrosia trifida* , variation in annual precipitation was a positive feedback on the mean effects of added NP, suggesting that the large gains in forb biomass and ANPP occurring with added NP and NPK were amplified by year‐to‐year variation in precipitation. Moreover, this suggests that precipitation mediates dominant species contribution to forb biomass, even if overall, forb biomass is unrelated to precipitation (Table 1, Briggs and Knapp 1995).

## 5 Conclusions

The core finding of this study is that N and P synergistically co‐limited ANPP, but Kμ interacted with N to reorder dominant species, driving responses in functional group biomass and species richness. These findings emphasize how adding different nutrients may limit different components of the community and highlight the key role of dominant species responses mediated by variation in annual precipitation. The findings highlight the need for an expanded definition of resource limitation in multispecies communities, accounting for differing limitations at species or functional group levels. This is important for refining models of productivity‐biodiversity relationships and may also have practical relevance. If the occurrence of positive diversity responses to added non‐limiting nutrients can be predicted, they may be a useful management tool for enhancing grassland biodiversity and biodiversity‐related ecosystem services. Clarifying how N, P, and K independently and jointly limit ecosystem productivity is critical to societal efforts to mitigate and adapt to the impacts of anthropogenic intensification of nutrient cycling on terrestrial ecosystem primary productivity and related ecosystem services (Sala et al. 2017; Migliavacca et al. 2021).

## Author Contributions

**Philip A. Fay:** conceptualization (lead), formal analysis (lead), project administration (lead), supervision (lead), writing – original draft (equal), writing – review and editing (equal). **Anita C. Risch:** conceptualization (supporting), funding acquisition (lead), writing – review and editing (supporting). **Michael J. Aspinwall:** project administration (supporting), writing – review and editing (supporting). **Robert W. Heckman:** project administration (supporting), writing – review and editing (supporting). **Albina R. Khasanova:** project administration (supporting), writing – review and editing (supporting). **Lara G. Reichmann:** project administration (supporting), writing – review and editing (supporting).

## Conflicts of Interest

The authors declare no conflicts of interest.

## Figures and Tables

**FIGURE A3:**
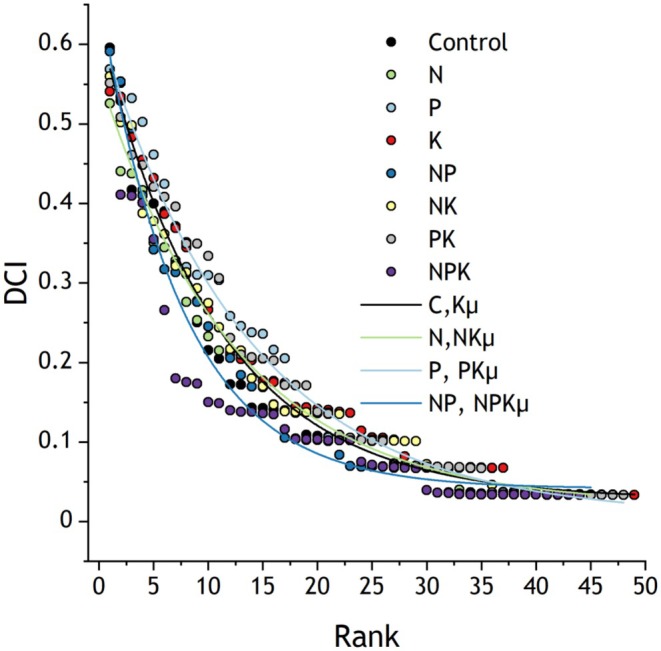
Rank abundance curves (Figure 4C) with data. Abundance values are the mean Dominance Candidate Index (DCI) for each species across years within treatments. Curves were fit to C, N, P, and NP treatments across levels of Kμ.

## Data Availability

Data and scripts are archived in Dryad. DOI: https://doi.org/10.5061/dryad.9zw3r22t0.

## Appendix A

**TABLE A1:** Species found across all plots and years of the study.

Taxon	Family	Functional group	Status	Lifespan	Class	DCI (±SE)	Cover (±SE)
*Ambrosia trifida*	Compositae	Forb	Native	Annual	Dominant	0.534 (0.016)	31.42 (2.58)
*Symphyotrichum ericoides*	Compositae	Forb	Native	Perennial	Dominant	0.456 (0.012)	20.88 (2.11)
*Schizachyrium scoparium*	Poaceae	Grass	Native	Perennial	Dominant	0.444 (0.013)	11.80 (1.17)
*Sorghum halepense*	Poaceae	Grass	Introduced	Perennial	Dominant	0.441 (0.014)	13.76 (1.88)
*Centaurea melitensis*	Compositae	Forb	Native	Annual	Dominant	0.411 (0.015)	9.87 (1.40)
*Monarda citriodora*	Lamiaceae	Forb	Native	Annual	Dominant	0.349 (0.018)	5.30 (0.57)
*Salvia azurea*	Lamiaceae	Forb	Native	Perennial	Dominant	0.305 (0.016)	7.86 (0.94)
*Andropogon gerardii*	Poaceae	Grass	Native	Perennial	Dominant	0.256 (0.015)	3.10 (0.46)
*Solanum dimidiatum*	Solanaceae	Forb	Native	Perennial	Subordinate	0.242 (0.015)	2.66 (0.34)
*Physalis mollis*	Solanaceae	Forb	Native	Perennial	Subordinate	0.208 (0.012)	2.74 (0.51)
*Panicum obtusum*	Poaceae	Grass	Native	Perennial	Subordinate	0.199 (0.008)	4.17 (0.69)
*Bouteloua curtipendula*	Poaceae	Grass	Native	Perennial	Subordinate	0.196 (0.015)	5.46 (1.02)
*Tragia brevispica*	Euphorbiaceae	Forb	Native	Perennial	Subordinate	0.195 (0.013)	2.93 (0.50)
*Oxalis stricta*	Oxalidaceae	Forb	Native	Perennial	Subordinate	0.177 (0.013)	2.16 (0.48)
*Rubus trivialis*	Rosaceae	Forb	Native	Perennial	Subordinate	0.165 (0.012)	5.20 (1.01)
*Trepocarpus aethusae*	Apiaceae	Forb	Native	Annual	Subordinate	0.159 (0.014)	3.22 (0.84)
*Limnodea arkansana*	Poaceae	Grass	Native	Annual	Subordinate	0.156 (0.018)	2.05 (0.48)
*Gaillardia pulchella*	Compositae	Forb	Native	Annual	Subordinate	0.153 (0.011)	4.88 (1.13)
*Stenaria nigricans*	Rubiaceae	Forb	Native	Perennial	Subordinate	0.149 (0.016)	3.74 (0.80)
*Psoralidium tenuiflorum*	Fabaceae	Legume	Native	Perennial	Subordinate	0.143 (0.011)	3.44 (0.72)
*Ruellia nudiflora*	Acanthaceae	Forb	Native	Perennial	Subordinate	0.137 (0.009)	1.08 (0.22)
*Galium texense*	Rubiaceae	Forb	Native	Annual	Subordinate	0.114 (0.015)	2.20 (0.76)
*Oenothera speciosa*	Onagraceae	Forb	Native	Perennial	Subordinate	0.103 (0.010)	1.16 (0.28)
*Polytaenia nuttallii*	Apiaceae	Forb	Native	Perennial	Subordinate	0.103 (0.012)	1.31 (0.34)
*Torilis arvensis*	Apiaceae	Forb	Introduced	Annual	Subordinate	0.102 (0.013)	2.26 (0.74)
*Physaria gracilis*	Brassicaceae	Forb	Native	Annual	Subordinate	0.098 (0.014)	0.83 (0.24)
*Brickellia eupatorioides*	Compositae	Forb	Native	Annual	Subordinate	0.073 (0.009)	0.65 (0.19)
*Nassella leucotricha*	Poaceae	Grass	Native	Perennial	Subordinate	0.069 (0.010)	0.62 (0.19)
*Sonchus asper*	Compositae	Forb	Introduced	Annual	Subordinate	0.068 (0.014)	0.36 (0.11)
*Sporobolus compositus*	Poaceae	Grass	Native	Perennial	Subordinate	0.064 (0.009)	0.64 (0.20)
*Glandularia bipinnatifida*	Verbenaceae	Forb	Native	Perennial	Subordinate	0.059 (0.010)	0.33 (0.13)
*Pyrrhopappus carolinianus*	Compositae	Forb	Native	Biennial	Subordinate	0.055 (0.012)	0.39 (0.16)
*Lactuca serriola*	Compositae	Forb	Introduced	Biennial	Subordinate	0.051 (0.009)	0.35 (0.13)
*Bifora americana*	Apiaceae	Forb	Native	Annual	Subordinate	0.050 (0.010)	1.65 (0.76)
*Sorghastrum nutans*	Poaceae	Grass	Native	Perennial	Subordinate	0.048 (0.008)	0.78 (0.33)
*Geranium* sp.	Geraniaceae	Forb	Native	Biennial	Subordinate	0.047 (0.008)	0.24 (0.08)
*Euphorbia spathulata*	Euphorbiaceae	Forb	Native	Annual	Subordinate	0.042 (0.010)	0.29 (0.14)
*Scutellaria drummondii*	Lamiaceae	Forb	Native	Annual	Subordinate	0.042 (0.007)	0.27 (0.11)
*Cirsium engelmannii*	Compositae	Forb	Native	Perennial	Subordinate	0.034 (0.007)	0.27 (0.11)
*Elymus canadensis*	Poaceae	Grass	Native	Perennial	Subordinate	0.030 (0.006)	0.18 (0.08)
*Physostegia virginiana*	Lamiaceae	Forb	Native	Perennial	Subordinate	0.030 (0.007)	0.28 (0.12)
*Triodanis perfoliata*	Campanulaceae	Forb	Native	Annual	Subordinate	0.030 (0.006)	0.36 (0.20)
*Ambrosia artemisiifolia*	Compositae	Forb	Native	Annual	Subordinate	0.028 (0.008)	1.14 (0.57)
*Daucus* sp.	Apiaceae	Forb	Native	Annual	Subordinate	0.027 (0.006)	1.18 (0.64)

*Note:* Dominance Candidate Index (DCI) and Cover values are means ± standard error (SE) averaged across all fertilizer treatments.

**TABLE A2:** Analysis of variance *F*‐statistics, degrees of freedom (dfs), and *p*‐values from linear mixed models fit to the aboveground net primary productivity (ANPP), forb and grass biomass, Shannon diversity index, Evenness, and Species Richness during 2008–2011 when previous years' standing dead biomass was not removed prior to the growing season.

Mixed model effects	ANPP		Forb		Grass		Shannon diversity		Evenness		Species Richness	
	*F* (dfs)	*p*	*F* (dfs)	*p*	*F* (dfs)	*p*	*F* (dfs)	*p*	*F* (dfs)	*p*	*F* (dfs)	*p*
N	0.1 (1, 16)	0.7872	4.8 (1, 16)	**0.0444**	1.2 (1, 16)	0.2855	0.5 (1, 16)	0.4923	0.3 (1, 16)	0.6180	2.6 (1, 16)	0.1249
P	0.1 (1, 16)	0.8029	0.3 (1, 16)	0.5938	0.6 (1, 16)	0.4470	1.8 (1, 16)	0.2041	0.6 (1, 16)	0.4471	5.9 (1, 16)	**0.0276**
N*P	0.1 (1, 16)	0.8073	0.0 (1, 16)	0.9599	7.2 (1, 16)	**0.0164**	0.8 (1, 16)	0.3844	0.1 (1, 16)	0.7319	0.0 (1, 16)	0.8905
K	0.0 (1, 16)	0.9047	1.3 (1, 16)	0.2729	0.0 (1, 16)	0.8699	5.4 (1, 16)	**0.0330**	1.9 (1, 16)	0.1888	2.7 (1, 16)	0.1183
N*K	0.5 (1, 16)	0.4722	3.8 (1, 16)	0.0689	0.9 (1, 16)	0.3532	0.1 (1, 16)	0.7684	0.5 (1, 16)	0.4923	1.1 (1, 16)	0.3157
P*K	0.5 (1, 16)	0.4843	0.1 (1, 16)	0.8125	0.0 (1, 16)	0.9182	2.1 (1, 16)	0.1664	0.7 (1, 16)	0.4064	0.1 (1, 16)	0.7177
N*P*K	1.0 (1, 16)	0.3292	4.4 (1, 16)	**0.0511**	0.3 (1, 16)	0.5658	0.0 (1, 16)	0.9672	0.0 (1, 16)	0.9430	0.5 (1, 16)	0.5056
